# Nutrient–response modeling with a single and interpretable artificial neuron

**DOI:** 10.1038/s41598-025-29267-w

**Published:** 2025-11-24

**Authors:** Hamed Ahmadi, Markus Rodehutscord

**Affiliations:** Institute of Animal Science, University of Hohenheim, Stuttgart, Germany

**Keywords:** Interpretable machine learning, Nutrient–response modeling, Parameter visualization, Computational biology and bioinformatics, Mathematics and computing

## Abstract

Precise estimation of nutrient requirements and utilization efficiency is fundamental to nutritional sciences, yet it is mainly performed using classical nonlinear regression models. These models are interpretable but require careful selection of the functional form and initial parameter values. Flexible machine learning (ML) methods are seemingly disliked due to their perceived “black box” nature, which can obscure biological insight. A minimal and interpretable ML framework addresses this gap in nutrient–response modeling. The proposed approach uses a single artificial neuron with a hyperbolic tangent activation. Mathematically, this resembles a four-parameter sigmoidal function but with greater flexibility and distinct parameter definitions, allowing capture of the monotonic, saturating dynamics typical of essential nutrient responses. The method is enhanced with modern ML best practices, including data augmentation, Bayesian regularization, and bootstrap resampling, providing robust, uncertainty-quantified estimates of key nutritional metrics—such as asymptotic response, inflection point, and nutrient requirements—even from small datasets. Evaluations across 12 diverse datasets from poultry and fish studies, including amino acids and phosphorus, demonstrated that the single artificial neuron matches or exceeds the performance of classical models while providing full analytical transparency. The framework is implemented as a no-code graphical application, ‘NutriCurvist’, offering an easy-to-use alternative tool for nutrient-response modeling to support data-driven, precision nutrition.

**Supplementary Information:**

The online version contains supplementary material available at 10.1038/s41598-025-29267-w.

## Introduction

Accurate estimation of both model parameters and derived nutritional metrics (e.g., nutrient requirements and utilization efficiencies) is central to precision nutrition. Nutrient–response (N–R) relationships describe how physiological processes such as growth and nutrient accretion scale with dietary nutrient supply. For decades, classical nonlinear regression models such as saturation kinetics, generalized saturation kinetics, logistic, exponential, and piecewise functions^1–5^ have been the standard for analyzing N–R data. These models are simple to execute with established computer programs and provide interpretable parameter estimates. However, they require choosing a functional form a priori and providing good initial parameter estimates. Moreover, their rigid structure can limit flexibility and robustness, particularly when dealing with small, noisy datasets, a common reality in controlled nutrition studies.

Machine learning (ML), particularly artificial neural networks (ANN), offers an alternative that can flexibly capture nonlinear dynamics without imposing rigid curve assumptions^6^. However, their adoption in many biological fields, specifically nutritional science, has been slow due to their reputation as “black boxes”: models whose internal mechanisms are hidden, making it difficult to extract biological meaning and interpret results, which reduces trust in hypothesis-driven studies^7^.

Recent advances in interpretable ML challenge the false dichotomy between flexibility and interpretability. Minimalist models that are “interpretable by design”^8,9^ may be inherently transparent, analytically tractable, and, in some cases, achieve performance comparable to more complex models. For instance, a single-neuron logistic model outperformed deep networks with thousands of parameters in predicting earthquakeaftershocks^10^, demonstrating that simplicity can rival complexity when thoughtfully matched to the problem structure. Such approaches align closely with the needs of nutrition science, where interpretation of estimated parameters is as important as predictive accuracy.

Based on the approach of using minimalist, interpretable ML models to bridge classical regression and ML in nutrition science, an interpretable ML framework for N–R curve modeling is proposed. The framework relies on a minimal ANN, referred to as a “*single artificial neuron*”, with a hyperbolic tangent (*tanh*) activation function. This model resembles a four-parameter sigmoidal function and captures the monotonic, saturating dynamics inherent to essential N–R while remaining fully transparent. Key nutritional metrics (e.g., requirements, inflection points, marginal utilization efficiency) are derived analytically from model parameters. To enhance robustness on small datasets, three ML best practices are integrated: controlled data augmentation via Gaussian noise to simulate biological variability^11,12^, Bayesian regularization to prevent overfitting and promote generalizable solutions^13^, and bootstrap resampling to generate rigorous, non-parametric uncertainty intervals for all derived metrics^14^.

We test this framework’s effectiveness across 12 diverse N–R datasets from poultry and fish studies, focusing on amino acids and phosphorus responses. To facilitate adoption, we implement the approach in ‘NutriCurvist’, a freely available, no-code graphical application that enables nutritionists to apply the framework without programming expertise.

## Methods

**Fig. 1:**
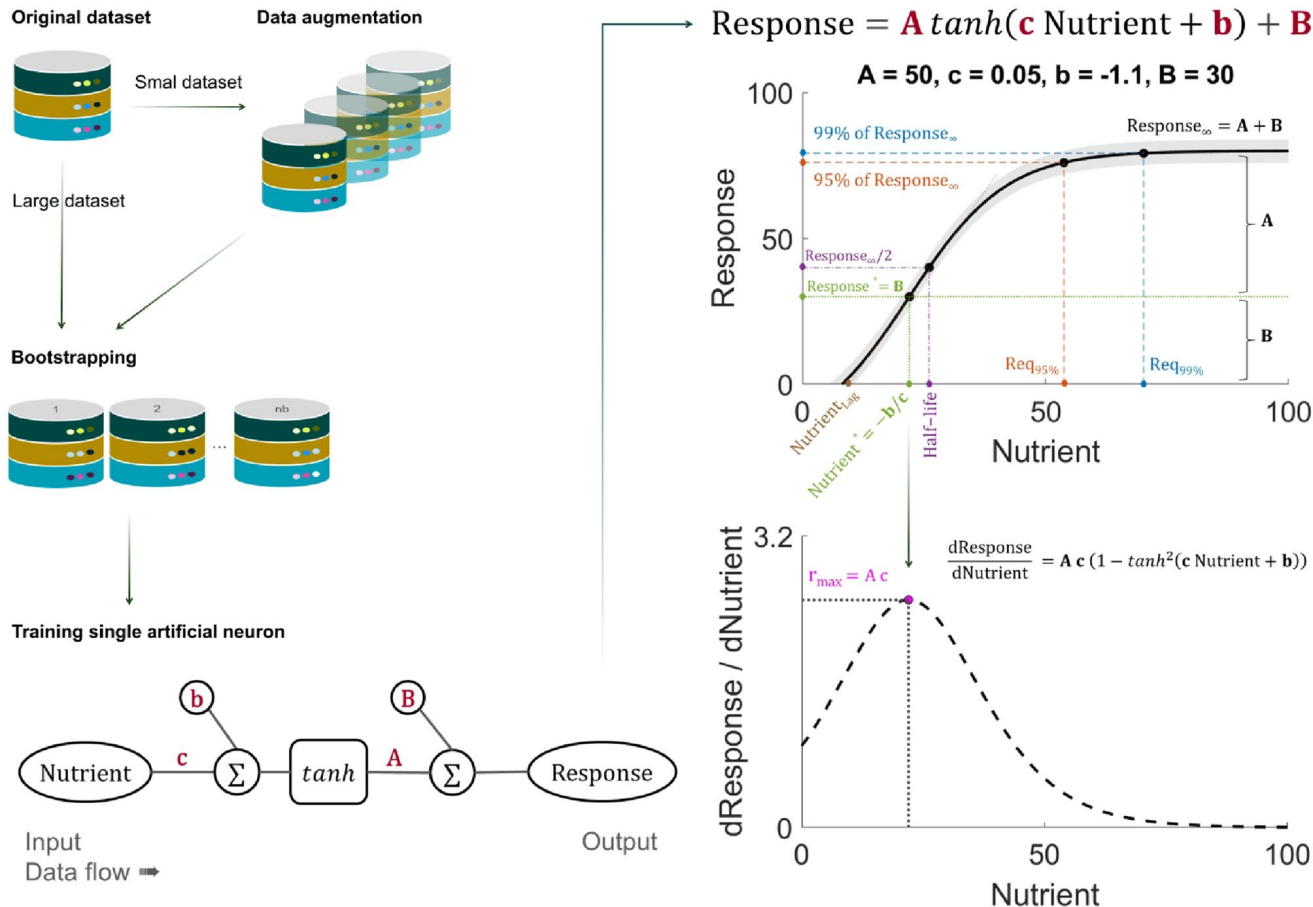
Framework of the nutrient–response (N–R) curve modeling using “single artificial neuron” model. The workflow begins with original datasets, where small datasets are augmented by adding Gaussian noise to generate synthetic replicates. Each augmented dataset is then resampled via bootstrapping to create robust training samples. A minimalist artificial neural network with one hidden neuron and hyperbolic tangent (*tanh*) activation is fitted to nutrient input and response output. The model is as simple as a four-parameter sigmoidal function, allowing direct derivation of key nutritional metrics from the fitted curves, including efficiency and requirement estimates. Response and first-derivative curves illustrate the N–R dynamics and rates of change, with confidence intervals obtained from bootstrap distributions.

At the core of our framework is a deliberately simplified ANN comprising a “*single artificial neuron*”, with a *tanh* activation function (Fig. 1). This architecture operates as a direct mathematical transformation from input nutrient levels to predicted biological response, defined by the equation:

1 $$\:\mathrm{R}\mathrm{e}\mathrm{s}\mathrm{p}\mathrm{o}\mathrm{n}\mathrm{s}\mathrm{e}\:=\:\mathrm{A}\:{tanh}\left(\mathrm{c}\:\mathrm{N}\mathrm{u}\mathrm{t}\mathrm{r}\mathrm{i}\mathrm{e}\mathrm{n}\mathrm{t}\:+\:\mathrm{b}\right)+\:\mathrm{B}$$

where Nutrient and Response are the input and output of the neuron, respectively; A, c, b, and B are trainable parameters estimated from the data (definition of parameters Table 1).

**Table 1:** Definition of “single artificial neuron” model, parameters, and derived nutritional metrics^1^.

	Explanation	Equation
Model	Nutrient-response curve describing how response changes with nutrient	$$\begin{gathered} \:{\mathrm{Response}}\: = \:{\mathrm{A}}\:tanh \hfill \\ \left( {{\mathrm{c}}\:{\mathrm{Nutrient}}\: + \:{\mathrm{b}}} \right) + \:{\mathrm{B}} \hfill \\ \end{gathered}$$
First derivative	Rate of change of the response with respect to nutrient	$$\begin{gathered} \:\frac{{{\mathrm{dResponse}}}}{{{\mathrm{dNutrient}}}} = {\mathrm{A}}\:{\mathrm{c}}\: \hfill \\ \left( {1 - tanh^{2} \left( {{\mathrm{c}}\:{\mathrm{Nutrient}} + {\mathrm{b}}} \right)} \right) \hfill \\ \end{gathered}$$
Parameters		
$\:\mathrm{A}$	Amplitude of the curve; determines how much the response increases beyond the inflection point	
$\:\mathrm{c}$	Steepness parameter: controls how quickly the response changes as nutrient level increases	
$\:\mathrm{b}$	Horizontal translation: shifts the curve left or right along the nutrient axis	
$\:\mathrm{B}$	Response at the inflection point; with B ≈ 0, the curve rises symmetrically from the origin toward the plateau	
Nutritional metrics		
$\:{\mathrm{r}}_{\mathrm{m}\mathrm{a}\mathrm{x}}$	Maximum slope of the curve; the steepest rate of increase in response per unit nutrient at the inflection point	$\:\mathrm{A}\:\:\mathrm{c}$
$\:{\mathrm{N}\mathrm{u}\mathrm{t}\mathrm{r}\mathrm{i}\mathrm{e}\mathrm{n}\mathrm{t}}^{\mathrm{*}}$	Nutrient level at the inflection point, where response changes most rapidly	$\:-\frac{\mathrm{b}}{\mathrm{c}}$
$\:{\mathrm{R}\mathrm{e}\mathrm{s}\mathrm{p}\mathrm{o}\mathrm{n}\mathrm{s}\mathrm{e}}^{\mathrm{*}}$	Response value at the inflection point (i.e. $\:{\mathrm{N}\mathrm{u}\mathrm{t}\mathrm{r}\mathrm{i}\mathrm{e}\mathrm{n}\mathrm{t}}^{\mathrm{*}}$)	$\:\mathrm{B}$
$\:{\mathrm{N}\mathrm{u}\mathrm{t}\mathrm{r}\mathrm{i}\mathrm{e}\mathrm{n}\mathrm{t}}_{\mathrm{L}\mathrm{a}\mathrm{g}}$	Lowest nutrient value where a meaningful response begins ($\:{\mathrm{R}\mathrm{e}\mathrm{s}\mathrm{p}\mathrm{o}\mathrm{n}\mathrm{s}\mathrm{e}}_{\mathrm{m}\mathrm{i}\mathrm{n}}=$ model-predicted response at lowest nutrient)	$$\begin{gathered} \:{\mathrm{Nutrient}}^{{\mathrm{*}}} \hfill \\ - \frac{{{\mathrm{Response}}^{{\mathrm{*}}} - {\mathrm{Response}}_{{{\mathrm{min}}}} }}{{{\mathrm{r}}_{{{\mathrm{max}}}} }} \hfill \\ \end{gathered}$$
$\:\mathrm{H}\mathrm{a}\mathrm{l}\mathrm{f}-\mathrm{l}\mathrm{i}\mathrm{f}\mathrm{e}$	Nutrient level at which 50% of the asymptotic response is reached	$\:\frac{{arctanh}\left(0.5-0.5\frac{\mathrm{B}}{\mathrm{A}}\:\right)-\:\mathrm{b}}{\mathrm{c}}$
$\:{\mathrm{R}\mathrm{e}\mathrm{s}\mathrm{p}\mathrm{o}\mathrm{n}\mathrm{s}\mathrm{e}}_{{\infty\:}}$	Asymptotic response as nutrient increases indefinitely	$\:\mathrm{A}\:+\:\mathrm{B}$
Requirement_95%_ ($\:{\mathrm{R}\mathrm{e}\mathrm{q}}_{95\mathrm{\%}}$)	Nutrient needed to achieve 95% of the $\:{\mathrm{R}\mathrm{e}\mathrm{s}\mathrm{p}\mathrm{o}\mathrm{n}\mathrm{s}\mathrm{e}}_{{\infty\:}}$	$\:\frac{{arctanh}\left(0.95-0.05\frac{\mathrm{B}}{\mathrm{A}}\:\right)-\:\mathrm{b}}{\mathrm{c}}$
Requirement_99%_ ($\:{\mathrm{R}\mathrm{e}\mathrm{q}}_{99\mathrm{\%}}$)	Nutrient needed to achieve 99% of the $\:{\mathrm{R}\mathrm{e}\mathrm{s}\mathrm{p}\mathrm{o}\mathrm{n}\mathrm{s}\mathrm{e}}_{{\infty\:}}$	$\:\frac{{arctanh}\left(0.99-0.01\frac{\mathrm{B}}{\mathrm{A}}\:\right)-\:\mathrm{b}}{\mathrm{c}}$

^1^Equations, parameters, and nutritional metrics described above are schematically illustrated in Fig. 1.

The computation proceeds as follows: The input nutrient value is multiplied by the weight parameter $\:\mathrm{c}$. The bias parameter $\:\mathrm{b}$ is added to form the linear combination, $\:\mathrm{z}\:=\:\mathrm{c}\:\times\:\:\mathrm{N}\mathrm{u}\mathrm{t}\mathrm{r}\mathrm{i}\mathrm{e}\mathrm{n}\mathrm{t}\:+\:\mathrm{b}$. This sum is passed through the *tanh* activation function, producing a smooth, bounded S-shaped curve ranging from − 1 to + 1. The result is scaled by the amplitude parameter $\:\mathrm{A}$ and shifted vertically by the parameter $\:\mathrm{B}$ to produce the final biological response.

This formulation resembles a four-parameter sigmoidal function but with greater flexibility and distinct parameter definitions, allowing it to capture the monotonic, saturating dynamics typical of essential N–R. Importantly, it is analytically tractable, allowing direct calculation of derivatives and derived nutritional metrics. The first derivative is given by the equation:

2 $$\:\frac{\mathrm{d}\mathrm{R}\mathrm{e}\mathrm{s}\mathrm{p}\mathrm{o}\mathrm{n}\mathrm{s}\mathrm{e}}{\mathrm{d}\mathrm{N}\mathrm{u}\mathrm{t}\mathrm{r}\mathrm{i}\mathrm{e}\mathrm{n}\mathrm{t}}=\mathrm{A}\:\mathrm{c}\:\left(1-{{tanh}}^{2}\left(\mathrm{c}\:\mathrm{N}\mathrm{u}\mathrm{t}\mathrm{r}\mathrm{i}\mathrm{e}\mathrm{n}\mathrm{t}+\mathrm{b}\right)\right)$$

This derivative represents the marginal efficiency of nutrient utilization, showing how much additional response is obtained per unit increase in nutrient. It reaches its peak when $\:\mathrm{c}\:\mathrm{N}\mathrm{u}\mathrm{t}\mathrm{r}\mathrm{i}\mathrm{e}\mathrm{n}\mathrm{t}+\mathrm{b}=0$. From the four model parameters, a series of nutritional metrics can be derived analytically (Fig. 1). The maximum rate of response change ($\:{\mathrm{r}}_{\mathrm{m}\mathrm{a}\mathrm{x}}$) occurs at the inflection point, defined by the nutrient level $\:{\mathrm{N}\mathrm{u}\mathrm{t}\mathrm{r}\mathrm{i}\mathrm{e}\mathrm{n}\mathrm{t}}^{\mathrm{*}}=-\frac{\mathrm{b}}{\mathrm{c}}$, where the corresponding response is $\:{\mathrm{R}\mathrm{e}\mathrm{s}\mathrm{p}\mathrm{o}\mathrm{n}\mathrm{s}\mathrm{e}}^{\mathrm{*}}=\mathrm{B}$. At this point, the slope equals $\:\mathrm{A}\:\mathrm{c}$, representing the highest marginal efficiency. The lag phase ($\:{\mathrm{N}\mathrm{u}\mathrm{t}\mathrm{r}\mathrm{i}\mathrm{e}\mathrm{n}\mathrm{t}}_{\mathrm{L}\mathrm{a}\mathrm{g}}$) indicates the earliest point at which supplementation produces a noticeable response, while the $\:\mathrm{h}\mathrm{a}\mathrm{l}\mathrm{f}-\mathrm{l}\mathrm{i}\mathrm{f}\mathrm{e}$ reflects the nutrient level at which the response reaches halfway between the baseline and the asymptotic response. The asymptotic response is given by $\:{\mathrm{R}\mathrm{e}\mathrm{s}\mathrm{p}\mathrm{o}\mathrm{n}\mathrm{s}\mathrm{e}}_{{\infty\:}}=\mathrm{A}+\mathrm{B}$, which represents the physiological ceiling under unlimited (infinity) nutrient supply. It should be noted that this framework does not capture potential reductions in response to excessive nutrient levels (e.g., toxic effects), which are beyond the scope of the current model. For practical feeding applications, requirement thresholds are defined as $\:{\mathrm{R}\mathrm{e}\mathrm{q}}_{95\mathrm{\%}}$ and $\:{\mathrm{R}\mathrm{e}\mathrm{q}}_{99\mathrm{\%}}$, corresponding to the nutrient levels needed to achieve 95% and 99% of $\:{\mathrm{R}\mathrm{e}\mathrm{s}\mathrm{p}\mathrm{o}\mathrm{n}\mathrm{s}\mathrm{e}}_{{\infty\:}}$ Together, these metrics translate the abstract model parameters into interpretable indicators of nutrient requirement and utilization efficiency. Formal definitions are provided in Table 1.

### Data augmentation

Following the idea established^11,15^ and practiced^12^ in low-data scientific ML, we integrated controlled data augmentation via multiplicative Gaussian noise. This is a principled regularization strategy that simulates the natural biological variability observed within experimental units to improve model generalizability without distorting the underlying data structure. We define “small data” as datasets with fewer than 5–10 unique observations (with or without replication per point) per N–R curve, typical of many nutrition experiments. For each original data point (Table 2), we generated 10 synthetic replicates by perturbing both the nutrient level (*x*) and response value (*y*) with Gaussian noise scaled proportionally to their magnitudes: Nutrient added noise = *N*(0, 0.02 × *x*); Response added noise = *N*(0, 0.05 × *y*). Importantly, these values are not fixed assumptions of the framework. In the final software implementation, both the number of synthetic replicates and the noise amplitudes are user-adjustable, allowing researchers to tailor them to the scale and variability of their specific dataset. Thus, the reported values are provided as default recommended settings that offer stable performance across typical nutrition datasets, rather than optimized or data-specific tuning choices.

**Table 2:** Summary of nutrient–response datasets used for model evaluation.

Dataset number	Species	Non-nutrient factor	Nutrient	Response(s)	Method used to model data	Summary of original results	References	Panel in Fig. 3
1	Broilers and males of layer strain	Species (broilers vs. layers)	Lysine (g/kg diet)	Body weight gain (g/bird)	Four-parameter logistic	Broilers: Response_∞_ = 534, Req_95%_ = 12.4, R^2^ = 0.96, Error = 43.1 Layers: Response_∞_ = 153, Req_95%_ = 10.1, R^2^ = 0.92, Error = 13.7	^18^	A
2	Broilers and males of layer strain	Species (broilers vs. layers)	Lysine (g/kg diet)	Feed efficiency (g/g)	Four-parameter logistic	Broilers: Response_∞_ = 0.820, Req_95%_ = 11.5, R^2^ = 0.87, Error = 0.07 Layers: Response_∞_ = 0.713, Req_95%_ = 11.1, R^2^ = 0.60, Error = 0.11	^18^	B
3	Broilers and males of layer strain	Species (broilers vs. layers)	Lysine (g/kg diet)	Protein accretion (g/bird)	Four-parameter logistic	Broilers: Response_∞_ = 87.1, Req_95%_ = 12.5, R^2^ = 0.95, Error = 7.98 Layers: Response_∞_ = 28.7, Req_95%_ = 10.4, R^2^ = 0.94, Error = 2.07	^18^	C
4	Broilers and males of layer strain	Species (broilers vs. layers)	Lysine (g/kg diet)	Lysine accretion (g/bird)	Four-parameter logistic	not reported in publication	^18^	D
5	Broilers and males of layer strain	Species (broilers vs. layers)	Lysine (g/kg diet)	Energy accretion (MJ/bird)	Four-parameter logistic	not reported in publication	^18^	E
6	Broilers and males of layer strain	Species (broilers vs. layers)	Lysine (g/kg diet)	Efficiency of energy utilization (%)	Four-parameter logistic	not reported in publication	^18^	F
7	Broiler chickens	Diets (LP; low-protein vs. NP; normal-protein)	Methionine (g/kg diet)	Protein accretion (g/bird)	Four-parameter logistic	LP diets: Response_∞_ = 86.5, Req_95%_ = 3.4, R^2^ = 0.94, Error = 6.57 NP diets: Response_∞_ = 82.1, Req_95%_ = 3.6, R^2^ = 0.94, Error = 7.18	^19^	G
8	White Pekin ducks	Age (week 1, 2, 3)	Valine (g/kg diet)	Body weight (g/duck)	Three-parameter exponential	Week 1: Response_∞_ = 288, Req_95%_ = 8.2, R^2^ = 0.90, Error = 11.3 Week 2: Response_∞_ = 832, Req_95%_ = 8.0, R^2^ = 0.84, Error = 31.6 Week 3: Response_∞_ = 1528, Req_95%_ = 7.9, R^2^ = 0.89, Error = 58.1	^20^	H
9	Broiler chickens		Valine (g/kg diet)	Weight gain (g)	Four-parameter logistic	Response_∞_ = 584.3, Req_95%_ = 7.9, R^2^ = 0.97, Error = 33.9	^21^	I
10	Laying hens		Threonine (mg/bird)	Egg mass (g)	Generalized logistic (T-model)	Response_∞_ = 49.2, Req_95%_ = 419, R^2^ = 0.78, Error = 3.07	^22^	J
11	Broilers, ducks, quails	Species (3 poultry species)	Phosphorus (g/kg diet)	Phosphorus accretion (mg/d)	Four-parameter logistic	Broilers: Response_∞_ = 340, Req_95%_ = 8.4, R^2^ = 0.92, Error = 21.7 Ducks: Response_∞_ = 606, Req_95%_ = 7.3, R^2^ = 0.95, Error = 34.6 Quails: Response_∞_ = 67.5, Req_95%_ = 4.8, R^2^ = 0.67, Error = 7.2	^23^	K
12	Rainbow trout (*O. mykiss*)		Phosphorus (g/kg diet)	Weight gain (g/fish)	Three-parameter exponential	Response_∞_ = 144, Req_95%_ = 3.7, R^2^ = 0.93, Error = 9.11	^24^	L

^1^Response_∞_ = asymptotic response as nutrient increases indefinitely; $\:{\mathrm{R}\mathrm{e}\mathrm{q}}_{95\mathrm{\%}}$ = nutrient needed to achieve 95% of the $\:{\mathrm{R}\mathrm{e}\mathrm{s}\mathrm{p}\mathrm{o}\mathrm{n}\mathrm{s}\mathrm{e}}_{{\infty\:}}$; Model error (Error) represents the RMSE (root mean square error between observed and predicted values), adjusted for the number of fitted parameters (3 or 4, depending on the model).

### Bootstrap uncertainty quantification

We implemented non-parametric bootstrap resampling (*n* = 100 iterations) to generate empirically asymmetric confidence intervals^14^ for all model parameters and derived nutritional metrics. This approach provides empirically grounded, asymmetric uncertainty bounds that are more reliable than asymptotic standard errors, especially for small datasets^16^. In each of 100 iterations, a bootstrap sample was drawn with replacement from the augmented dataset, matching the size of the original dataset. This simulates the sampling variability inherent in repeated experiments. After 100 iterations, the distribution of each parameter and metric was summarized by its mean and 95% confidence interval (CI), defined as the 2.5th and 97.5th percentiles. This yields asymmetric, data-driven uncertainty bounds that reflect the true variability in the estimates without assuming normality or large-sample behavior. This setting is intended to provide stable CIs and consistent error values while keeping computation times reasonable for our investigated datasets. However, in the final software, the number of bootstrap iterations can be adjusted by the user depending on dataset size and desired precision, as more iterations may narrow CIs but increase computation time. The default setting of 100 iterations is therefore recommended as a practical balance rather than a dataset-specific optimization.

**Fig. 2:**
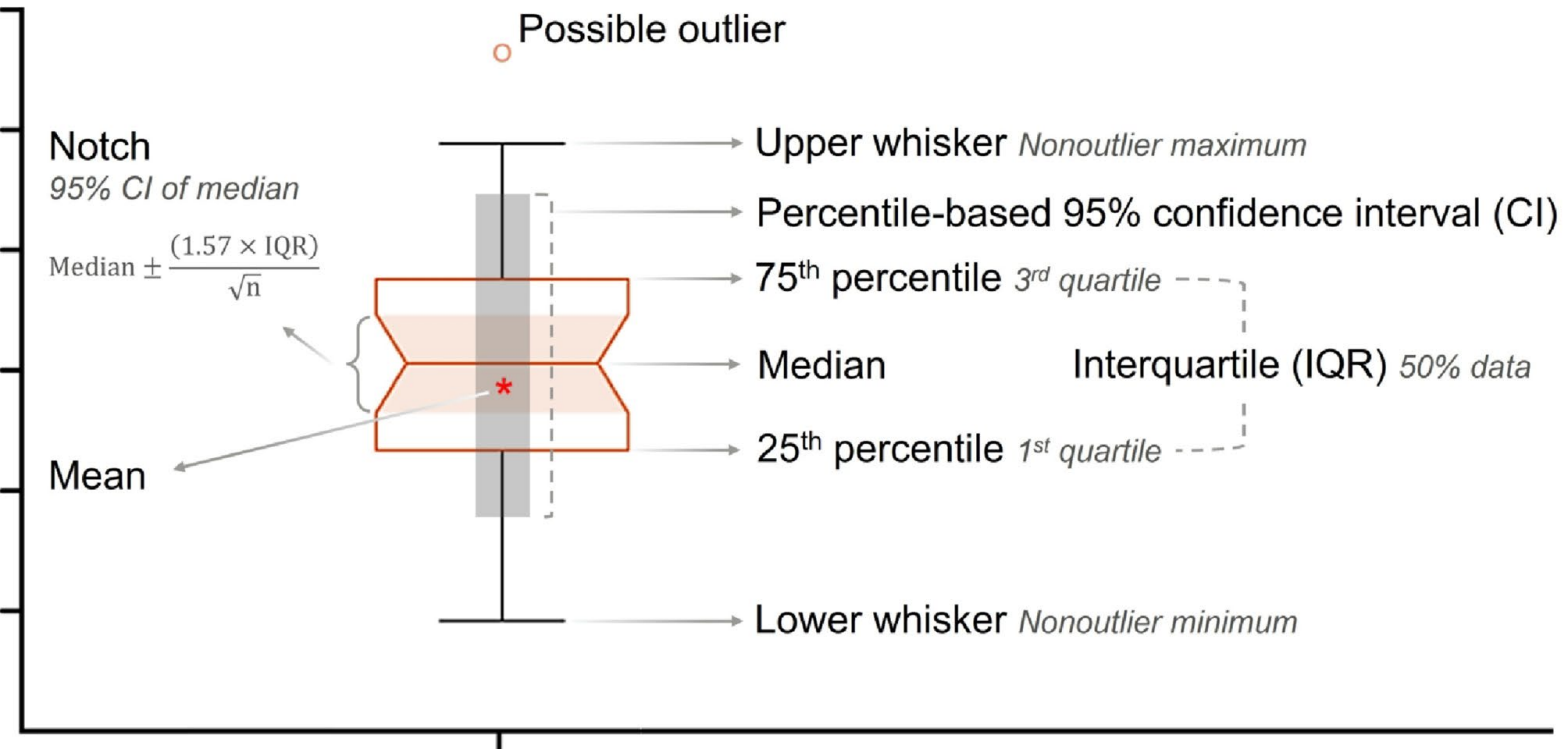
Schematic representation of an enhanced box plot. The central box shows the interquartile range (IQR; 25th–75th percentile) with the median indicated by the horizontal line. Whiskers extend to the minimum and maximum non-outlier values, while possible outliers are plotted individually. The notch represents an approximate 95% confidence interval (CI) of the median, where non-overlapping notches suggest significant median differences between groups^17^. In addition, the mean (red asterisk) and its bootstrap-derived 95% CI (grey rectangle; 100 iterations) are displayed to provide the primary metric of interest, reflecting both central tendency and empirical uncertainty. While notches serve as a visual guide, all formal statistical inferences in this study are based on one-way ANOVA applied to the bootstrap distributions, followed by Tukey’s honest significant difference (HSD) test for multiple comparison of means.

The “selected model” reported in tables and figures (e.g., Table 3; Fig. 2) corresponds to the single bootstrap iteration with the lowest root mean square error (RMSE) relative to the mean prediction across all 100 models. This ensures reported values are representative of the most accurate fit while remaining embedded within the full uncertainty distribution.

**Table 3:** Summary of nutrient–response model equations, fitted parameters, and derived nutritional metrics across diverse species, nutrients, and responses^1^.

Information on data					Fitted model	Nutritional metrics								Model parameters				Goodness of fit	
Dataset number	Species	Non-nutrient factor	Nutrient	Response	Equation	r_max_	Nutrient^*^	Response^*^	Nutrient_Lag_	Half-life	Response_∞_	Req_95%_	Req_99%_	A	c	b	B	R^2^	Error
1	Broilers	Broilers	Lys (g/kg diet)	BWG (g/bird)	BWG = 235.9 *tanh*(0.510 Lys − 4.34) + 297.0	120.5 (113.5, 130.7)	8.48 (8.35, 8.59)	296.4 (289.1, 303.3)	6.55 (6.42, 6.68)	8.22 (8.1, 8.32)	532.2 (515.5, 545.6)	11.2 (10.9, 11.5)	12.9 (12.4, 13.2)	235.9 (227.6, 242.7)	0.51 (0.478, 0.557)	-4.34 (-4.69, -4.07)	297 (289.1, 303.6)	0.961	39.8
1	Layers	Layers	Lys (g/kg diet)	BWG (g/bird)	BWG = 57.9 *tanh*(0.462 Lys − 3.11) + 96.2	27.3 (24.2, 31.4)	6.72 (6.33, 7.08)	96.6 (92.4, 101.3)	4.83 (4.42, 5.34)	5.96 (5.68, 6.25)	153.9 (150.9, 157.2)	9.5 (9.12, 9.8)	11.3 (10.6, 11.8)	57.9 (53.4, 61.5)	0.462 (0.406, 0.586)	-3.11 (-4.09, -2.64)	96.2 (92.4, 101.4)	0.913	13.1
2	Broilers	Broilers	Lys (g/kg diet)	FE (g/g)	FE = 0.237 *tanh*(0.389 Lys − 2.93) + 0.588	0.092 (0.081, 0.104)	7.49 (7.07, 7.85)	0.586 (0.562, 0.606)	5.19 (4.59, 5.7)	4.99 (4.6, 5.35)	0.823 (0.807, 0.839)	10.5 (10.1, 11.1)	12.7 (12, 13.7)	0.237 (0.219, 0.262)	0.389 (0.316, 0.453)	-2.93 (-3.52, -2.24)	0.588 (0.561, 0.606)	0.886	0.062
2	Layers	Layers	Lys (g/kg diet)	FE (g/g)	FE = 0.169 *tanh*(0.431 Lys − 3.25) + 0.545	0.073 (0.056, 0.106)	7.47 (6.4, 8.22)	0.541 (0.505, 0.569)	5.24 (4.04, 6.63)	3.59 (3.59, 3.59)	0.718 (0.701, 0.74)	10.3 (9.63, 11.1)	12.4 (11.1, 13.9)	0.169 (0.155, 0.208)	0.431 (0.28, 0.641)	-3.25 (-5.24, -1.84)	0.545 (0.505, 0.569)	0.632	0.081
3	Broilers	Broilers	Lys (g/kg diet)	PA(g/bird)	PA = 38.21 *tanh*(0.509 Lys − 4.38) + 48.8	19.6 (18.3, 21.4)	8.6 (8.44, 8.71)	48.7 (47.2, 50.1)	6.68 (6.52, 6.81)	8.33 (8.19, 8.43)	86.9 (84.1, 89.5)	11.3 (11, 11.7)	13 (12.5, 13.4)	38.2 (36.9, 39.5)	0.509 (0.475, 0.563)	-4.38 (-4.81, -4.08)	48.8 (47.2, 50.1)	0.950	7.30
3	Layers	Layers	Lys (g/kg diet)	PA (g/bird)	PA = 10.4 *tanh*(0.470 Lys − 3.22) + 18.6	4.85 (4.36, 5.62)	6.84 (6.6, 7.08)	18.5 (17.9, 19.2)	4.9 (4.54, 5.36)	5.95 (5.72, 6.16)	28.9 (28.5, 29.5)	9.64 (9.23, 9.99)	11.4 (10.7, 12.1)	10.4 (9.82, 11)	0.47 (0.396, 0.57)	-3.22 (-4.02, -2.64)	18.6 (17.9, 19.2)	0.939	1.99
4	Broilers	Broilers	Lys (g/kg diet)	LA (g/bird)	LA = 2.72 *tanh*(0.478 Lys − 4.25) + 3.21	1.32 (1.24, 1.42)	8.89 (8.76, 9.01)	3.21 (3.13, 3.29)	6.86 (6.76, 7)	8.71 (8.57, 8.83)	5.93 (5.77, 6.08)	11.8 (11.5, 12.1)	13.6 (13.1, 13.9)	2.72 (2.65, 2.79)	0.478 (0.456, 0.526)	-4.25 (-4.66, -4.08)	3.21 (3.13, 3.29)	0.968	0.417
4	Layers	Layers	Lys (g/kg diet)	LA (g/bird)	LA = 0.641 *tanh*(0.412 Lys − 2.84) + 0.928	0.261 (0.235, 0.296)	6.87 (6.62, 7.12)	0.923 (0.891, 0.967)	4.72 (4.39, 5.15)	6.33 (6.15, 6.51)	1.57 (1.54, 1.6)	10.3 (9.82, 10.7)	12.3 (11.6, 13.1)	0.641 (0.611, 0.686)	0.412 (0.347, 0.469)	-2.84 (-3.35, -2.31)	0.928 (0.891, 0.967)	0.950	0.108
5	Broilers	Broilers	Lys (g/kg diet)	EnA (MJ/bird)	EnA = 2.15 *tanh*(0.486 Lys − 4.20) + 2.66	1.04 (0.97, 1.13)	8.65 (8.53, 8.77)	2.65 (2.59, 2.71)	6.61 (6.46, 6.77)	8.4 (8.28, 8.51)	4.8 (4.68, 4.9)	11.6 (11.2, 11.9)	13.3 (12.8, 13.8)	2.15 (2.09, 2.2)	0.486 (0.441, 0.531)	-4.2 (-4.54, -3.85)	2.66 (2.59, 2.71)	0.967	0.329
5	Layers	Layers	Lys (g/kg diet)	EnA (MJ/bird)	EnA = 0.431 *tanh*(0.401 Lys − 2.48) + 0.629	0.174 (0.163, 0.189)	5.99 (5.26, 6.68)	0.601 (0.507, 0.675)	4.13 (3.71, 4.71)	5.59 (5.33, 5.91)	1.06 (1.04, 1.09)	9.73 (9.26, 10.2)	11.9 (11, 13)	0.431 (0.394, 0.557)	0.401 (0.302, 0.492)	-2.48 (-3.21, -1.59)	0.629 (0.507, 0.675)	0.898	0.096
6	Broilers	Broilers	Lys (g/kg diet)	EnE (%)	EnE = 12.30 *tanh*(0.377 Lys − 2.97) + 29.1	4.59 (3.71, 5.57)	7.85 (7.45, 8.19)	28.9 (27.4, 30.1)	5.37 (4.6, 6.04)	5.69 (5.37, 6.07)	41.5 (40.8, 42.3)	11.2 (10.5, 12.1)	13.6 (12.4, 15.2)	12.3 (11.4, 13.7)	0.377 (0.269, 0.466)	-2.97 (-3.77, -2.01)	29.1 (27.4, 30.1)	0.862	3.56
6	Layers	Layers	Lys (g/kg diet)	EnE (%)	EnE = 11.4 *tanh*(0.224 Lys − 1.10) + 16.2	2.54 (2, 3.45)	5.09 (3.59, 7.28)	16.5 (12.6, 20.6)	3.75 (3.59, 4.65)	3.82 (3.59, 4.17)	27.7 (27.1, 28.6)	11.1 (9.93, 12.5)	14.8 (12.6, 16.9)	11.4 (6.83, 16.5)	0.224 (0.179, 0.311)	-1.097 (-2.27, -0.632)	16.2 (10.8, 20.6)	0.623	3.20
7	Broilers	Low protein (LP) diets	Met (g/kg diet)	PA (g/bird)	PA = 32.4 *tanh*(1.91 Met − 5.14) + 54.6	62.8 (56.4, 70.2)	2.69 (2.64, 2.74)	54.4 (52.8, 56.4)	2.19 (2.11, 2.29)	2.5 (2.47, 2.54)	86.9 (85.4, 88.3)	3.37 (3.3, 3.48)	3.8 (3.67, 3.99)	32.4 (30.8, 35)	1.91 (1.65, 2.25)	-5.15 (-6.12, -4.41)	54.6 (52.7, 56.4)	0.944	6.12
7	Broilers	Normal protein (NP) diets	Met (g/kg diet)	PA (g/bird)	PA = 32.0 *tanh*(2.19 Met − 6.21) + 50.0	68.3 (60.3, 78.9)	2.83 (2.79, 2.86)	49.9 (48.7, 51.1)	2.37 (2.3, 2.43)	2.7 (2.66, 2.72)	82 (80.6, 83.8)	3.47 (3.35, 3.56)	3.86 (3.68, 4)	32 (31.2, 33.4)	2.19 (1.88, 2.51)	-6.21 (-7.07, -5.32)	50 (48.7, 51.1)	0.940	6.62
8	Ducks	Week 1	Val (g/kg diet)	Body weight (g/duck)	BW = 56.4 *tanh*(1.42 Val − 10.4) + 229.3	78.6 (69.7, 94.5)	7.31 (7.02, 7.57)	225.9 (208.8, 238.1)	6.69 (6.45, 6.98)	6.37 (6.37, 6.37)	285.5 (283.1, 287.8)	8.07 (7.93, 8.19)	8.73 (8.46, 8.98)	56.4 (45.8, 77.2)	1.42 (1.02, 1.8)	-10.44 (-13.4, -7.08)	229.3 (208.7, 238)	0.921	10.0
8	Ducks	Week 2	Val (g/kg diet)	Body weight (g/duck)	BW = 117.6 *tanh*(1.97 Val − 14.79) + 712.2	245.6 (184.3, 330.6)	7.5 (7.15, 7.66)	711.5 (646.8, 733.2)	7.1 (6.85, 7.34)	6.83 (6.83, 6.83)	829 (822.3, 835.5)	7.88 (7.8, 8)	8.32 (8.1, 8.57)	117.6 (95.5, 182.8)	1.97 (1.34, 3.34)	-14.79 (-25.6, -9.51)	712.2 (646.6, 732.9)	0.863	25.2
8	Ducks	Week 3	Val (g/kg diet)	Body weight (g/duck)	BW = 238.8 *tanh*(2.40 Val − 18.1) + 1283.4	579.4 (477.7, 738.7)	7.49 (7.26, 7.66)	1265 (1182.8, 1315.3)	7.09 (6.86, 7.34)	6.78 (6.78, 6.78)	1524.8 (1512, 1536.2)	7.9 (7.82, 8)	8.3 (8.15, 8.5)	238.8 (209.4, 342.5)	2.4 (1.57, 3.12)	-18.1 (-23.9, -11.4)	1283.4 (1183.2, 1314.9)	0.910	46.8
9	Broilers	Broilers	Val (g/kg diet)	Weight gain (g)	WG = 236.1 *tanh*(1.10 Val − 7.23) + 348.9	257 (232.8, 280.6)	6.56 (6.47, 6.62)	345.7 (330.6, 356.6)	5.79 (5.7, 5.88)	6.35 (6.31, 6.39)	585.5 (577.5, 592.1)	7.84 (7.69, 8)	8.62 (8.37, 8.88)	236.1 (228.4, 255.8)	1.1 (0.934, 1.22)	-7.23 (-7.96, -6.07)	348.9 (330.2, 356)	0.965	32.0
10	Layers	Layers	Thr (mg/bird)	EM (g)	EM = 33.2 *tanh*(0.006 Thr − 0.945) + 17.1	0.191 (0.172, 0.219)	170 (147.6, 207.2)	18.2 (13.6, 24.5)	148.2 (147.6, 150.7)	206.2 (202.5, 209.8)	50.3 (49.8, 50.9)	443 (416.2, 473.8)	584.1 (533.7, 642.6)	33.2 (25.2, 47.2)	0.006 (0.005, 0.007)	-0.945 (-1.44, -0.49)	17.1 (3.56, 24.4)	0.801	2.59
11	Broilers	Broilers	Phosphorus (g/kg diet)	PhoA (mg/d)	PhoA = 236.9 *tanh*(0.303 Phosphorus − 0.797) + 97.6	72.4 (66.8, 79.3)	2.83 (2.75, 3.19)	111.3 (102.2, 137.1)	2.75 (2.75, 2.76)	3.63 (3.54, 3.73)	334.7 (324, 346.2)	8.08 (7.45, 8.73)	10.8 (9.62, 11.9)	236.9 (190.7, 312.8)	0.303 (0.258, 0.372)	-0.797 (-1.17, -0.457)	97.6 (23.2, 137)	0.917	20.0
11	Ducks	Ducks	Phosphorus (g/kg diet)	PhoA (mg/d)	PhoA = 301.8 *tanh*(0.412 Phosphorus − 1.68) + 310.7	124.9 (120, 131.7)	4.06 (3.75, 4.33)	308.4 (272.8, 336.7)	2.9 (2.82, 3.03)	4.05 (3.97, 4.13)	612.9 (591, 631.1)	7.65 (7.1, 8.36)	9.67 (8.76, 10.8)	301.8 (261.8, 355.3)	0.412 (0.342, 0.503)	-1.68 (-2.13, -1.31)	310.7 (272.8, 336.6)	0.959	30.0
11	Quails	Quails	Phosphorus (g/kg diet)	PhoA (mg/d)	PhoA = 33.1 *tanh*(0.755 Phosphorus − 2.06) + 33.8	24.8 (20.8, 29.7)	2.91 (2.79, 3.42)	38 (34, 48.2)	2.8 (2.79, 2.9)	2.79 (2.79, 2.79)	66.8 (65.8, 68.1)	4.62 (4.31, 4.96)	5.66 (4.98, 6.34)	33.1 (17.6, 46)	0.755 (0.597, 1.28)	-2.06 (-4.38, -1.41)	33.8 (21.9, 48.2)	0.671	5.75
12	Rainbow trout	Rainbow trout	Phosphorus (g/kg diet)	Weight gain (g/fish)	WG = 57.5 *tanh*(1.17 Phosphorus − 2.12) + 86.8	68.9 (59.4, 78.1)	1.81 (1.7, 1.9)	87 (81.7, 90.6)	1.19 (1.09, 1.3)	1.59 (1.54, 1.64)	144.3 (141.8, 146.4)	2.95 (2.76, 3.19)	3.65 (3.34, 4.07)	57.5 (52.5, 64.9)	1.17 (0.959, 1.47)	-2.12 (-2.75, -1.62)	86.8 (81.8, 90.6)	0.954	7.07

^1^Nutritional metrics are averaged over all bootstrap iterations (*n* = 100), and parameter values are taken from the selected model per dataset, identified as the one with the lowest error relative to the average prediction across all bootstrap models. Values in parentheses represent 95% confidence intervals (2.5th–97.5th percentiles) of the bootstrap distributions, allowing for asymmetric bounds. Model error is expressed as RMSE (root mean square error between observed and predicted values from the selected model). Analyses were based on measured values plus 10 augmented variants per point generated by adding 2 and 5% Gaussian noise to the nutrient and response values, respectively. Lys, lysine; Met, methionine; Val, valine; Thr, threonine; BWG, body weight gain; BW, body weight; WG, weight gain; FE, feed efficiency; PA, protein accretion; LA, lysine accretion; EnA, energy accretion; EnE, efficiency of energy utilization; PhoA, phosphorus accretion. For parameter and metric definitions, see Table 1. For data sources, see Table 2.

### Bayesian regularization

Model training employed Bayesian regularization algorithm using MATLAB’s ‘*trainbr’*, which automatically balances fit accuracy and parameter smoothness by penalizing large weights (MATLAB^®^ Version 24.2, R2024b, The MathWorks Inc.). This prevents overfitting on small datasets, promotes generalizable solutions, and stabilizes convergence^13^ for robust ANN training under data scarcity. While *trainbr* is computationally slower than some other training algorithms, the combination of 10 synthetic replicates per original data point and 100 bootstrap iterations produced stable parameter estimates with reasonable computation time (~ 30 s per dataset on a standard desktop).

### Visualization and statistical comparisons across datasets

To better visualize the estimated parameters and nutritional metrics, we created box plots^17^ with several enhancements (Fig. 2). Each plot shows the interquartile range, median, whiskers, and potential outliers, with notches indicating the approximate 95% CI of the median. In addition, the mean and its bootstrap-derived 95% CI were included as the primary metric of interest, reflecting both central tendency and empirical uncertainty. Non-overlapping notches between two box plots provide a quick visual indication of significant median differences. However, for deeper and statistically robust comparisons of parameters and nutritional metrics across species, diets, or nutrients, we performed one-way ANOVA on the full bootstrap distributions (*n* = 100 values per metric per dataset), followed by Tukey’s Honest Significant Difference (HSD) post hoc tests to identify pairwise differences among means at *p* < 0.05.

### Data sources and N–R datasets

To evaluate the versatility and reliability of the proposed modeling framework, 12 independent N–R datasets (Table 2) were analyzed, encompassing four species [chicken (broilers, layers), Pekin ducks, Japanese quail, rainbow trout], five nutrients (lysine, methionine, valine, threonine, phosphorus), and diverse response variables (body weight gain, feed efficiency, protein/lysine/phosphorus accretion, energy accretion, egg mass, weight gain), enabling assessment of the modeling framework under diverse dietary, physiological, and experimental conditions. Six datasets were derived from lysine response trials in broilers and males of a layer strain, assessing body weight gain, feed efficiency, protein accretion, lysine accretion, energy accretion, and efficiency of energy utilization^18^. One dataset described methionine-driven protein accretion in broilers^19^. One dataset originated from valine body weight response trials in White Pekin ducks^20^. One dataset involved valine-driven weight gain in broiler chickens^21^. One dataset comprised a compiled meta-analysis of threonine intake–egg mass responses in laying hens^22^. One dataset described phosphorus response in poultry (chickens, ducks, and quails), measuring phosphorus accretion under graded dietary phosphorus supplementation^23^. One dataset described phosphorus response in rainbow trout, assessing weight gain under graded inclusion of phosphorus in a semi purified diet^24^. All datasets were obtained in raw format from original experiments.

## Results

### Model performance

The single artificial neuron model provided robust and stable fits across all datasets. As summarized in Table 3, the model achieved goodness-of-fit metrics, with R^2^ and RMSE values within acceptable ranges. When compared with the regression models reported in the original studies (for datasets where such results were available; see Table 2), the single artificial neuron attained an average R^2^ of 0.89 (range 0.63–0.97) and an average RMSE of 11.40 (range 0.06–46.9), compared with 0.88 (range 0.60–0.97) and 17.14 (range 0.07–58.1) for the original models. Although maximizing goodness-of-fit was not the primary aim here, our approach performed equivalently or better than the reference models. This outcome is encouraging, as it demonstrates that the model balances accuracy and interpretability, providing a reliable foundation for subsequent derivation of nutritional metrics.

### Derived nutritional metrics

**Fig. 3:**
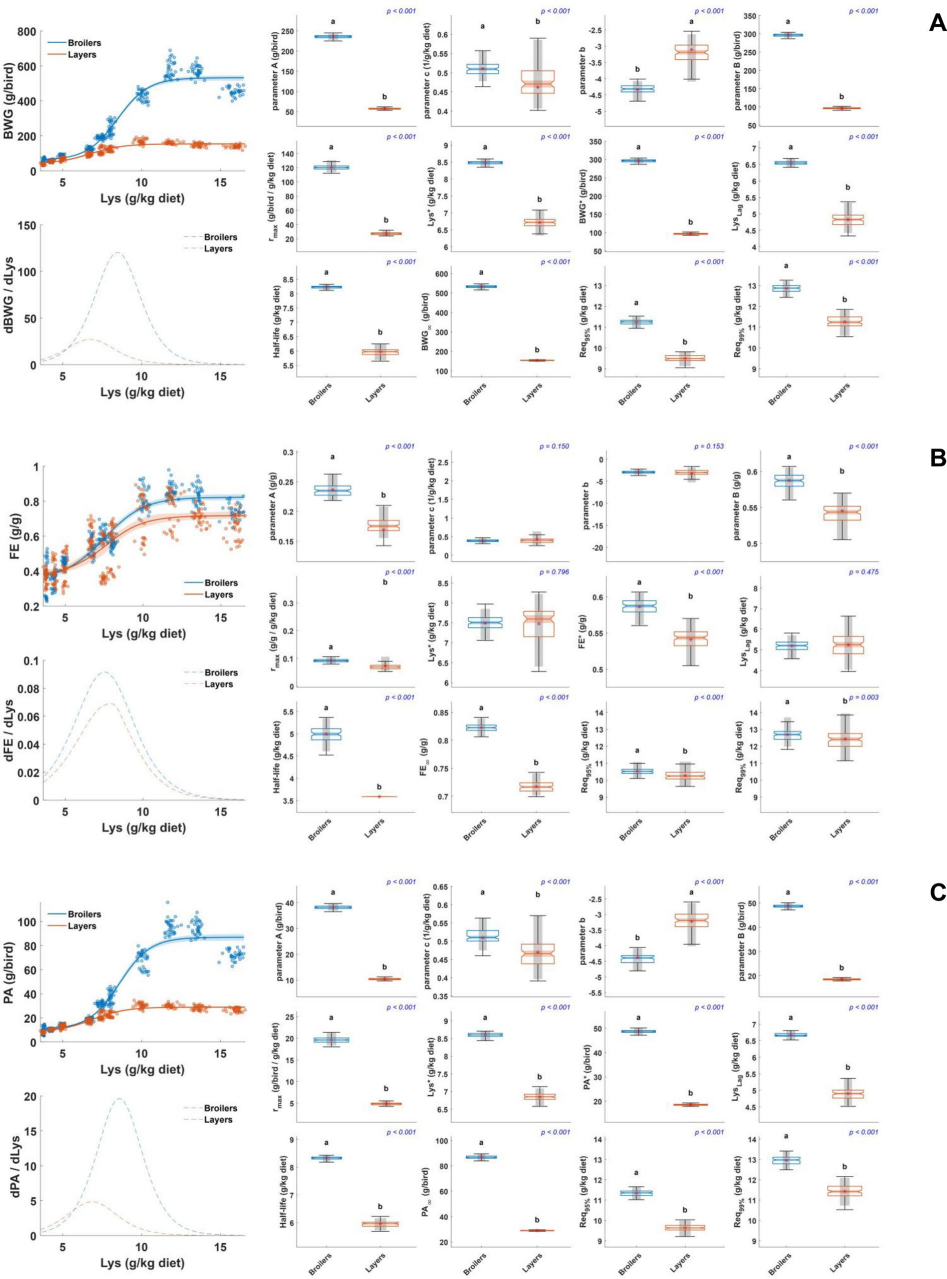

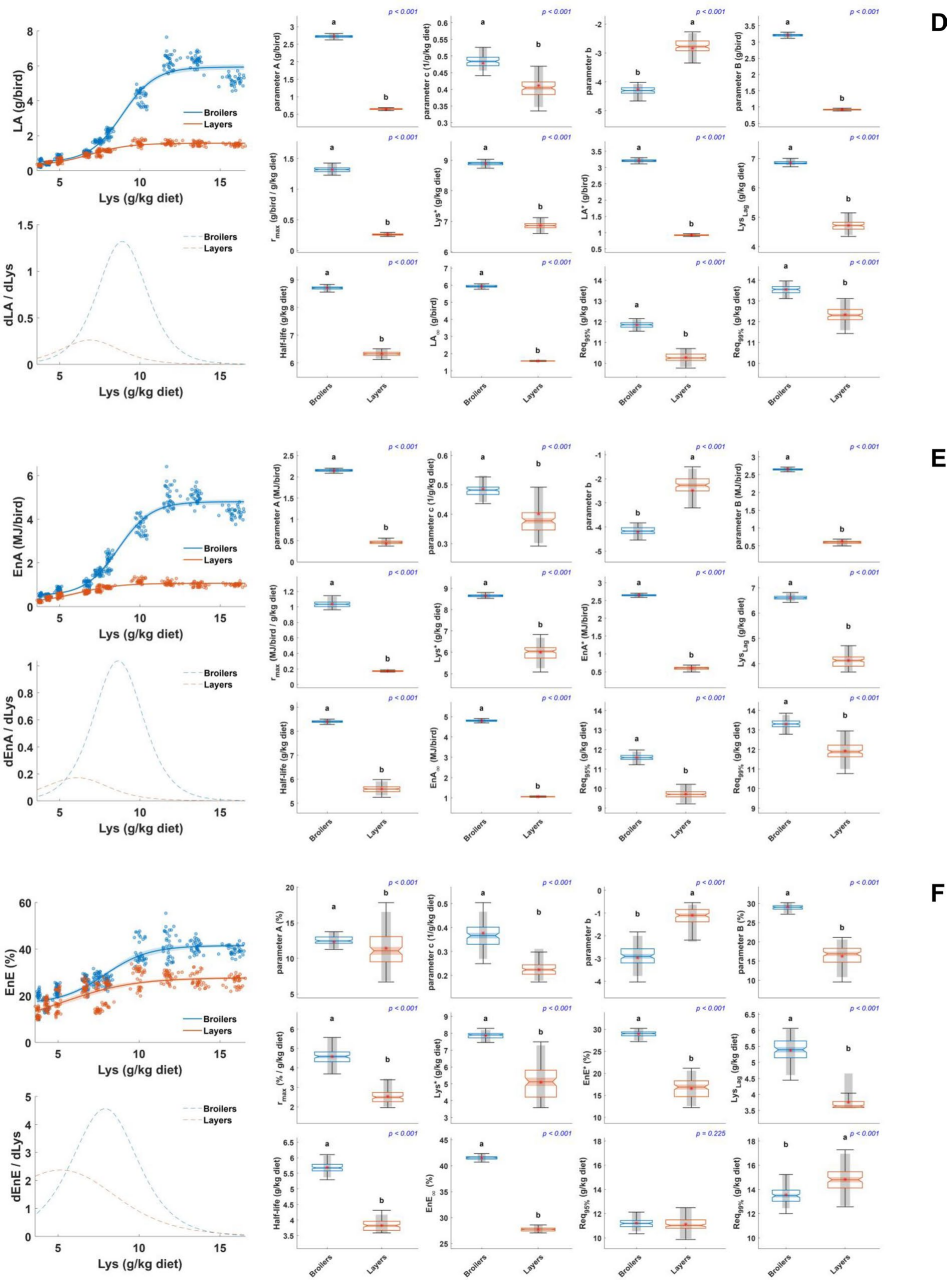

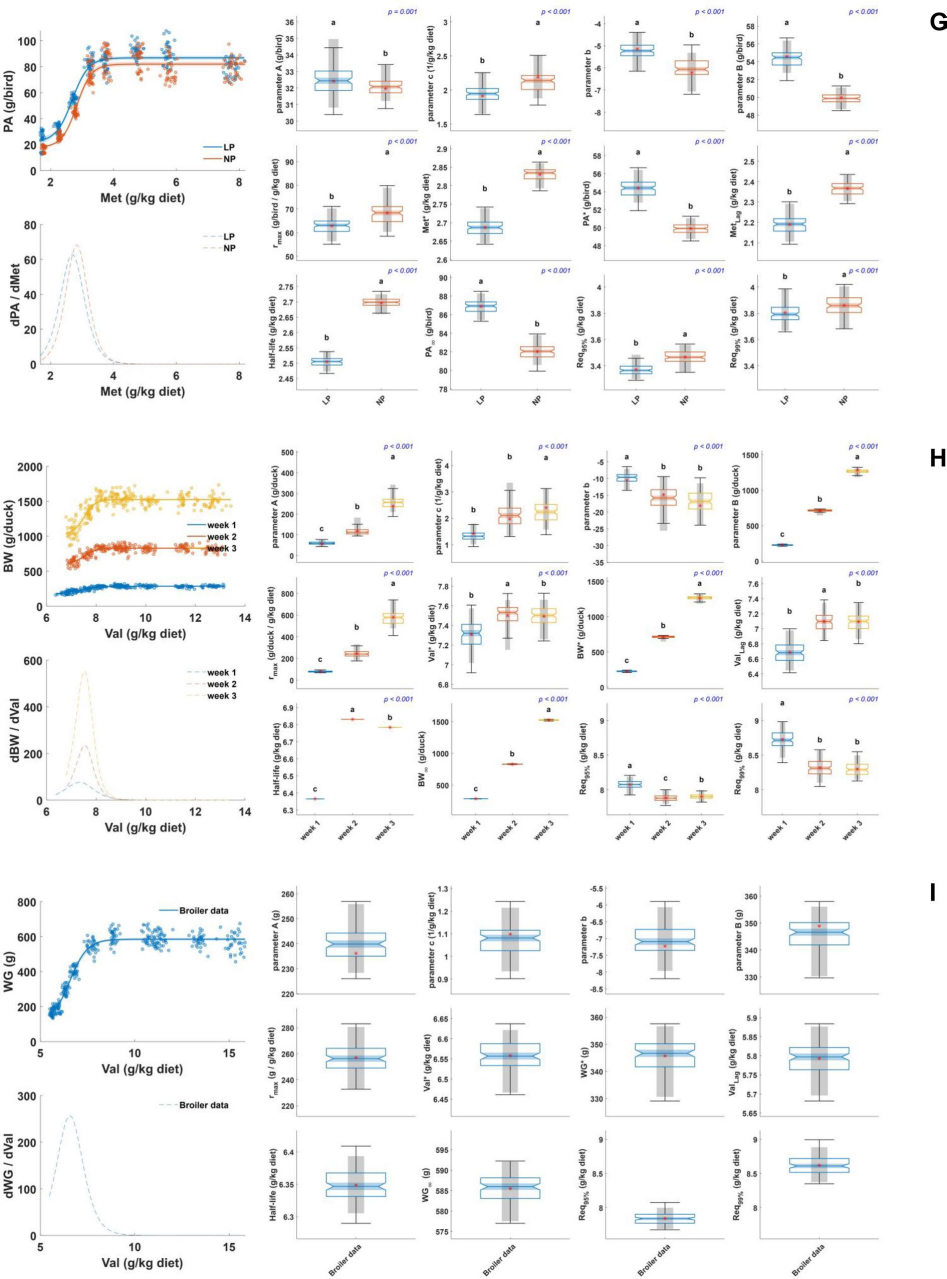

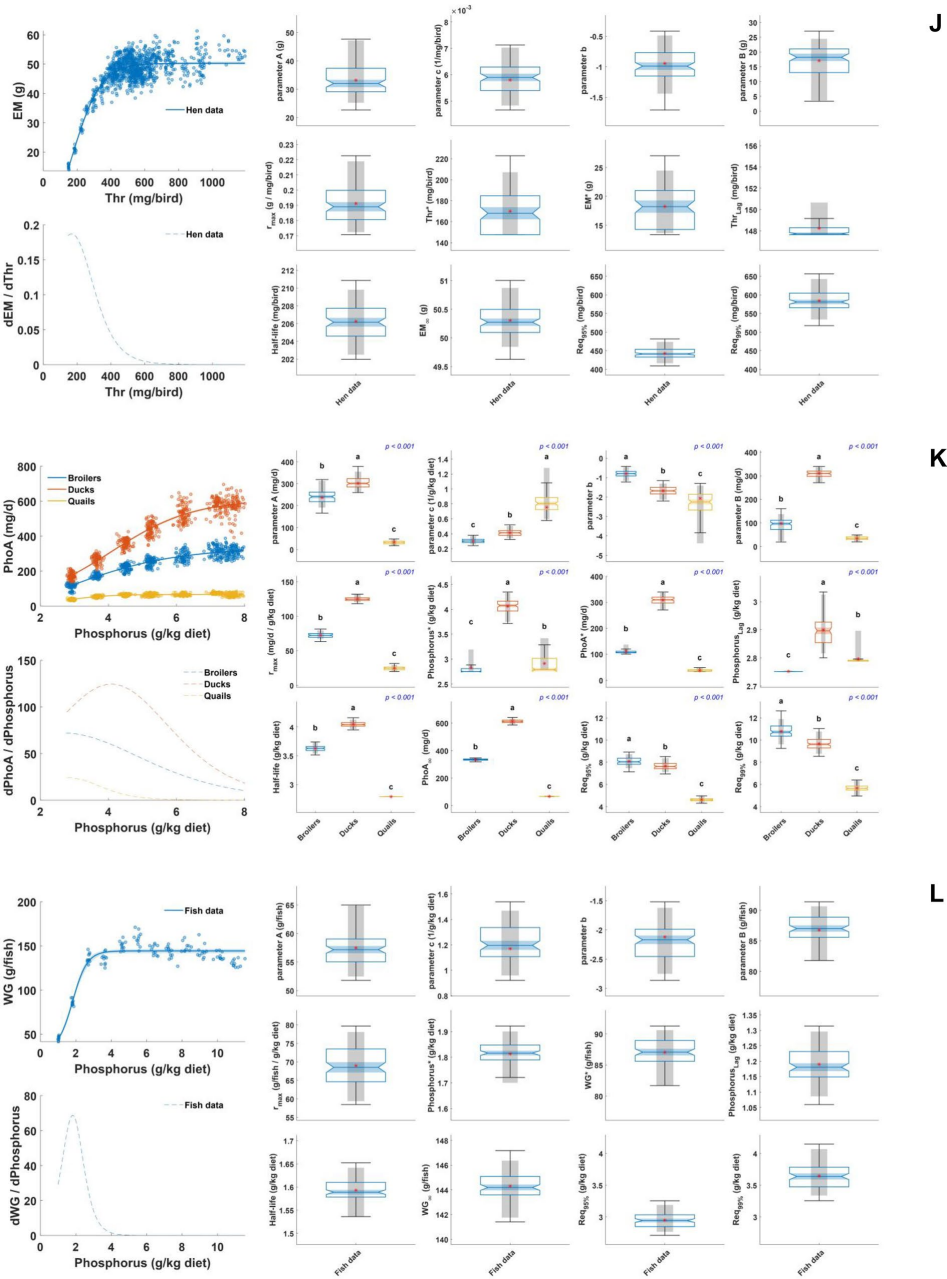
Nutrient–response relationships modeled across datasets. Responses of growth, nutrient accretion, and efficiency metrics to dietary nutrients across multiple studies. Panels (**A**–**F**) show lysine (Lys) responses in male broilers and layers from 8 to 21 days: (**A**) body weight gain (BWG), (**B**) feed efficiency (FE), (**C**) protein accretion (PA), (**D**) lysine accretion (LA), (**E**) energy accretion (EnA), and (**F**) efficiency of energy (EnE). Panel (**G**) depicts protein accretion (PA) response to dietary methionine (Met) in broilers fed low-protein (LP; 183 g CP/kg) and normal-protein (NP; 229 g CP/kg) diets. Panel (**H**) illustrates body weight (BW) responses to dietary valine (Val) in male ducks from three different ages growing for a 3-week period. Panel (**I**) shows weight gain (WG) response to dietary valine (Val) in male broiler chickens. Panel (**J**) shows egg mass (EM) response to dietary threonine (Thr) intake in laying hens (compiled from 9 experiments). Panel (**K**) shows phosphorus accretion (PhoA) responses in three bird species (broilers, ducks, quails) between days 21 and 25 of age. Panel (**L**) depicts weight gain response to dietary phosphorus in rainbow trout over 53 days. Data points represent measured responses per study from multiple pens or observations, plus 10 augmented variants per point generated by adding Gaussian noise equal to 2 and 5% of the original nutrient and response values, respectively. Solid curves depict NutriCurvist model fits. First-derivative plots illustrate the marginal rate of change in each response across amino acid levels. Bar panels display extracted nutritional metrics and model parameters from the selected model per genotype, diet, age, or response type, averaged over all bootstrap iterations. Models were selected based on the lowest error relative to the average prediction of all bootstrap models. Error bars indicate 95% confidence intervals derived from the 2.5th–97.5th percentiles of the bootstrap distributions, allowing for asymmetric bounds. Statistical annotations report p-values and compact letters from one-way ANOVA with Tukey’s HSD (*p* < 0.05) applied to the full bootstrap distributions of each metric and parameter. Original data sources for each panel are provided in Table 2.

Derived nutritional metrics across all datasets are summarized in Table 3. The response curves exhibited S-shaped, monotonic, and diminishing-return behavior in all cases, as evidenced by the $\:{\mathrm{N}\mathrm{u}\mathrm{t}\mathrm{r}\mathrm{i}\mathrm{e}\mathrm{n}\mathrm{t}}^{\mathrm{*}}$ values (Table 3) and first-derivative plots (Fig. 3). The first derivative of the response with respect to nutrient level remained positive across the observed range but declined toward zero as the nutrient supply approached the requirement range, indicating a progressively slower increase in response. The peak of the derivative corresponds to the maximum marginal efficiency $\:{\mathrm{r}}_{\mathrm{m}\mathrm{a}\mathrm{x}}$, while the decline toward zero reflects the approach to $\:{\mathrm{R}\mathrm{e}\mathrm{s}\mathrm{p}\mathrm{o}\mathrm{n}\mathrm{s}\mathrm{e}}_{{\infty\:}}$. Bootstrap-derived 95% CI around $\:{\mathrm{r}}_{\mathrm{m}\mathrm{a}\mathrm{x}}$ further supported this pattern, showing that the lower bounds of the slope remained positive (significantly different from zero).

Derived $\:{\mathrm{R}\mathrm{e}\mathrm{s}\mathrm{p}\mathrm{o}\mathrm{n}\mathrm{s}\mathrm{e}}_{{\infty\:}}$ (Table 3) closely matched the reported values (Table 2), with ranges of 0.719–1524 versus 0.713–1528, respectively. Estimated $\:{\mathrm{R}\mathrm{e}\mathrm{q}}_{95\mathrm{\%}}$ was generally consistent with values reported by the original regression models, with ranges of 2.85–439.3 versus 3.4–419 for the single artificial neuron and reference models, respectively. Relative differences between single artificial neuron and original $\:{\mathrm{R}\mathrm{e}\mathrm{q}}_{95\mathrm{\%}}$ values mainly were within ± 5%. Slightly larger deviations were observed for datasets 1–3, where single artificial neuron estimates were approximately 9% lower than the original values, and for dataset 12 (rainbow trout, phosphorus), where the difference reached ~ 23%. This dataset contained no replication points, and the original study used a three-parameter exponential model, which lacks a true inflection point and transitions abruptly from baseline to the asymptote, explaining the larger discrepancy. For all other datasets, including multi-week duck trials and multi-species phosphorus experiments, single artificial neuron-derived $\:{\mathrm{R}\mathrm{e}\mathrm{q}}_{95\mathrm{\%}}$ (Table 3) matched the values reported in the original regression models (Table 2).

Overall, single artificial neuron-derived $\:{\mathrm{R}\mathrm{e}\mathrm{q}}_{95\mathrm{\%}}$ values tended to be slightly lower than the original model estimates. Exceptions were observed for dataset 10 (laying hen threonine, compiled from publications) and dataset 11 (duck phosphorus response), where single artificial neuron estimates were approximately 5% higher than the original values.

### Inter-dataset comparison

Datasets 1–8 and 11 included a non-nutrient factor, enabling comparisons of N–R curves and derived nutritional metrics across these factors. Boxplots in Fig. 3A–H,K visually summarize these comparisons. Differences between groups were assessed both visually, using the notches in the boxplots—which approximate 95% CIs around the median—and statistically, using compact letter displays derived from bootstrap-based ANOVA followed by Tukey HSD tests. It should be noted that the Tukey HSD here assumes a normal distribution of the bootstrap samples and treats each bootstrap replicate as an observation; therefore, this is not a classical Tukey test in the experimental sense. Nevertheless, agreement between notch non-overlap in the boxplots and the Tukey results supports the reliability of group comparisons.

For datasets 1–6, which included both broilers and males of a layer strain under comparable dietary treatments^18^, the majority of parameter estimates and derived nutritional metrics (≈ 93%) differed significantly between strains (*P* < 0.05). This demonstrates that both the shape of the N–R curves and the resulting nutritional requirements are strongly strain-dependent. For instance, in dataset 1, broilers exhibited higher values of parameters A, c, |b|, and B, as well as consistently greater derived metrics, reflecting their higher growth potential and greater lysine requirement.

Dataset 7, which tested protein accretion (PA) response to dietary methionine (Met) in broiler chickens fed low-protein (LP; 183 g CP/kg) and normal-protein (NP; 229 g CP/kg) diets^19^, revealed clear differences (*P* < 0.001) in Met response dynamics between the two dietary treatments. Although both diets supported substantial PA, the LP diet achieved a higher $\:{\mathrm{P}\mathrm{A}}_{{\infty\:}}$, whereas the NP diet required higher Met levels to reach 95% and 99% of $\:{\mathrm{P}\mathrm{A}}_{{\infty\:}}$ (*P* < 0.001). These results indicate that PA was more efficient under LP feeding, with birds reaching near-maximal growth at lower Met levels.

This type of interpretation, supported by the statistical outcomes of our method, can in principle be applied to all other datasets that include non-nutrient factors. However, since a detailed discussion of each parameter and metric across all datasets lies beyond the scope of this study, we did not provide dataset-by-dataset interpretations.

## Discussion

### Integrating interpretability with flexibility and accuracy

We employed a single artificial neuron with a *tanh* activation to balance biological plausibility, interpretability, and parameter count. Since N–R relationships are monotonic and saturating, properties are well captured by the bounded S-shape of *tanh*. Unlike deeper networks, the single-neuron design yields a closed-form four-parameter equation in which each parameter maps directly to a meaningful quantity, preserving nutritional insight. Consistent with the principle that simplicity can rival complexity^8,10^, additional network depth may provide only marginal R² improvements while widening CIs, reducing interpretability, and increasing sensitivity to initialization. The *tanh* function was chosen over alternatives such as, linear, polynomial or logistic sigmoid functions because it enforces asymptotic behavior, offers symmetric saturation that supports mechanistic interpretations of saturation and inhibition^25^, and provides greater numerical stability during optimization^26^. By restricting the ANN architecture to a single neuron with a *tanh* activation, we retained the flexibility of nonlinear approximation while preserving full interpretability of model parameters. This contrasts with classical regression functions, which require predefined curve selection, and deep learning models, which are often criticized as “black box”. Our results demonstrate that interpretability and flexibility are not mutually exclusive: a simple ANN can yield biologically meaningful descriptors while maintaining robust statistical properties.

### Comparison with classical regression models

Unlike standard nonlinear regression, which requires predefined initial parameter values and focuses primarily on curve fitting, the proposed single-neuron formulation constrains the model structure such that the fitted parameters maintain direct nutritional meaning. This design enables transparent interpretation of saturation behavior while preserving the computational simplicity of a minimal neural network. Across the datasets tested, our approach matched or exceeded the performance of logistic or exponential regressions originally applied in the single studies. Importantly, our approach provided derivative-based metrics, such as maximum slope and marginal efficiency curves, which are not usually available from classical regression models. A key advantage of our framework is that all estimated and derived metrics are accompanied by bootstrap-derived CI. This offers a practical assessment of uncertainty and reliability, going beyond simple point estimates or averages^16^. Moreover, unlike classical regression, particularly four-parameter models, our framework incorporates automated parameter search and regularization to identify stable parameter estimates without requiring manual initialization. Recent tools^27^ similarly implement automated initialization for tanh-type models. However, our approach differs in that parameter exploration and stabilization occur within the training procedure itself, removing the need for user expertise or iterative trial-and-error tuning, which is often a limiting factor when applying nonlinear regression to small datasets. These features make the approach robust and user-friendly, enabling consistent application across diverse datasets and experimental conditions; however, in terms of computation time, classical nonlinear regression is faster.

### Robustness under practical data limitations

A persistent challenge in nutrition research is the small size and heterogeneity of experimental datasets, which can lead to convergence difficulties and unstable parameter estimates, particularly when initial values are uncertain. In our framework, controlled data augmentation increases the effective data density around measured points, reducing the risk of overfitting and improving numerical stability in the estimation of the first derivative. This is complemented by Bayesian regularization, which discourages over-parameterization, and non-parametric bootstrapping, which quantifies uncertainty without assuming normality. Together, these components produced stable parameter estimates and relatively narrow confidence intervals, even under small-sample conditions (Table 3; Fig. 3).

This percentage-based perturbation preserves the heteroscedastic nature of biological data—larger responses naturally show greater variability—while maintaining biologically plausible noise. For example, ± 5% perturbation on a 500 g body weight gain simulates ± 25 g variation, consistent with typical coefficients of variation in poultry trials, and ± 2% noise on 10 g/kg lysine introduces ± 0.2 g/kg variation, within assay precision limits^18,21^. The approach is theoretically justified in the equivalence between training with noise and regularization^9^, which promotes smoother, generalizable fits. Small proportional Gaussian perturbations have also enhanced robustness without bias in low-data domains^12^. Importantly, augmentation was applied before bootstrap resampling, so each iteration trained on a slightly different, biologically realistic version of the dataset. This two-stage process allowed us to stabilize parameter estimates across a broader simulated distribution and empirically quantify uncertainty through non-parametric bootstrap distributions reflecting both noise measurement and biological variability.

The framework performed reliably even in datasets without replication, such as Dataset 12, which included only 12 measured responses against nutrient level, and Dataset 10, which was constructed as a meta-dataset from nine studies. Although the latter was not heterogeneous in design, it still demonstrated that the single-neuron framework could generate consistent and interpretable results. These examples highlight the robustness of the approach across both minimal and composite datasets, turning data scarcity into a tractable modeling condition. From a methodological standpoint, this robustness is a critical advantage: it reduces dependence on user expertise in model initialization and allows the same framework to be applied consistently across species, nutrients, and response variables. From a nutritional standpoint, it enables more confident inference from limited experimental resources—a situation common in animal science, where studies are costly and ethically constrained.

### Supporting comparative and integrative analyses

The bootstrapped single artificial neuron framework allows direct statistical comparison of nutritional metrics within (and across) datasets. This extends beyond simple N–R curve fitting, enabling the extraction of broader insights from the same experimental data. For example, in the present work, our method enabled meaningful comparisons of how lysine responses and requirements differ between broilers and males of a layer strain (datasets 1–6; Table 3; Fig. 3A–F), how protein accretion in broiler chickens responds to dietary Met under low- vs. normal-protein diets (dataset 7; Table 3; Fig. 3G), how ducks of different ages grow (body weight) in response to dietary valine (dataset 8; Table 3; Fig. 3H), and to what extent the response to phosphorus varies among broilers, ducks, and quails (dataset 11; Table 3; Fig. 3K). These comparisons were statistically supported by bootstrap-derived CIs and compact letter displays. Group-level differences were evaluated using a combination of graphical and statistical criteria. Visual inspection of boxplots provided approximate CI around the medians, while bootstrap-based ANOVA with Tukey-type post hoc comparisons offered a complementary statistical assessment. Although these post hoc comparisons rely on assumptions about the bootstrap distributions and are not identical to classical experimental Tukey tests, their consistency with the graphical evidence lends confidence to the robustness of the group comparisons.

The ability to perform such inter-dataset evaluations is particularly relevant for precision nutrition, where integrative meta-analyses are increasingly valued. Importantly, this framework also facilitates hypothesis-driven testing in nutrition, addressing common research questions such as whether species, genotypes, or dietary treatments differ significantly in their nutrient requirements. In this way, the approach not only characterizes response patterns but also provides a decision-support tool for requirement evaluation and comparative nutrition research.

### Accessibility through a graphical user interface (GUI)

**Fig. 4:**
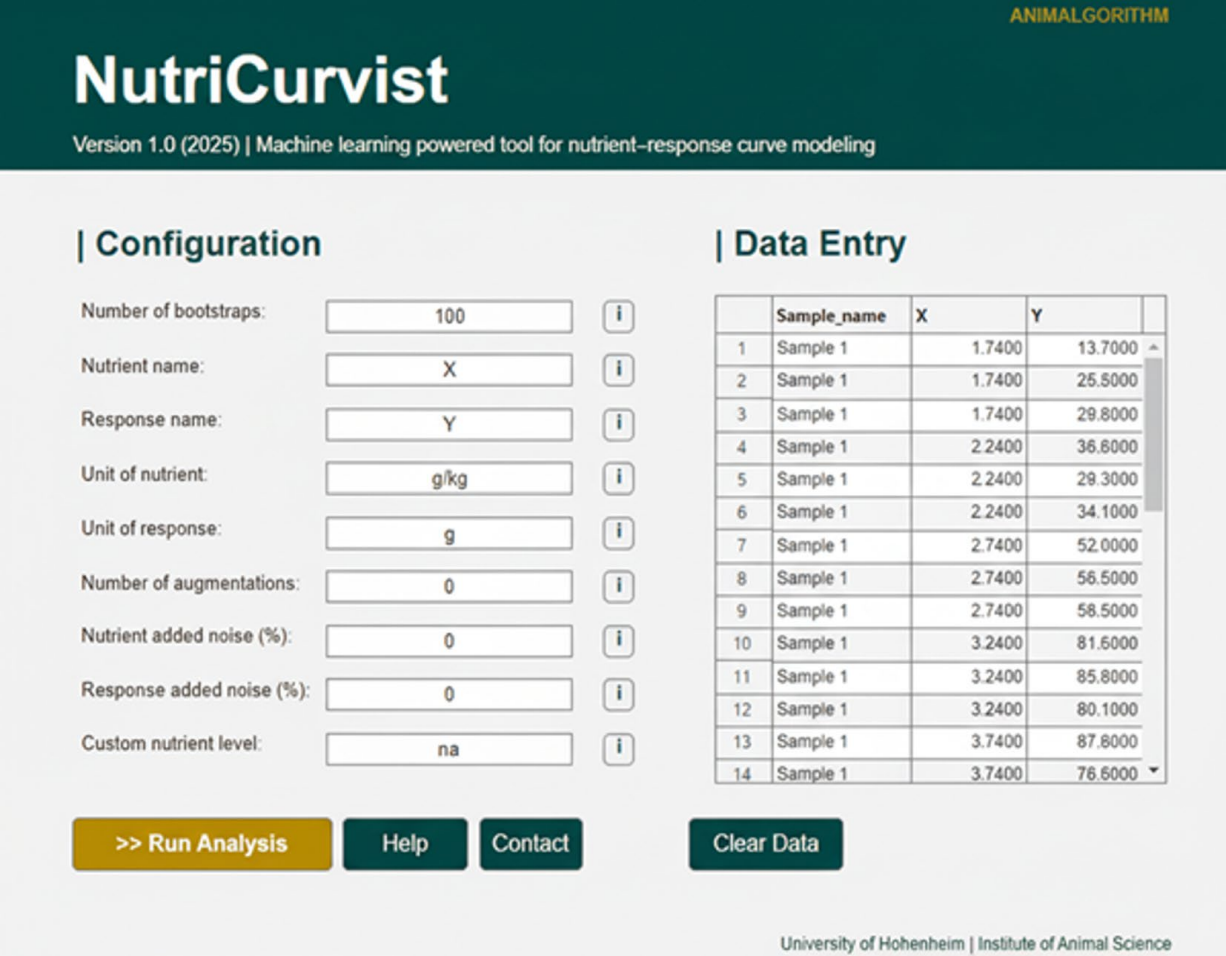
User interface of the NutriCurvist application. Screenshot of the graphical interface developed for nutrient–response curve modeling using *“single artificial neuron”* model. Users can import data, configure model settings, visualize response curves with uncertainty bands, and export nutritional metrics without requiring programming knowledge.

In many studies, ML models cannot be readily reused by end users due to the complexity of the algorithms and the need for specialized software or programming skills. To overcome this barrier, we developed a GUI for our single-neuron framework, called NutriCurvist (Fig. 4), which requires no programming expertise. This design enables researchers to import data, fit models, and export figures through an intuitive interface, facilitating the broader adoption of interpretable ML tools in applied nutrition. NutriCurvist also adopts FAIR-inspired principles^28^ by being openly Findable, Accessible, Interoperable, and Reusable with standard data formats, promoting reproducibility and standardization across nutritional studies. Instructions for installing and using NutriCurvist software are provided in Supplementary File 1. The main characteristics of the single artificial neuron algorithm implemented in NutriCurvist are summarized in Table 4.

**Table 4:** Characteristics of “*single artificial neuron*” modeling framework implemented in NutriCurvist.

Characteristic	Description
Model type	Single artificial neuron (1-hidden neuron feedforward neural network)
Activation function	Hyperbolic tangent (*tanh*)
Main and auxiliary algorithms	Feedforward neural network trained via backpropagation with Bayesian Regularization; Data Augmentation; Bootstrapping
Complexity	Minimalist; comparable to a 4-parameter logistic model; without unnecessary complexity
Interpretability	Both parameters and derivatives are nutritionally interpretable
Range of application	Handles monotonic nutrient–response relationships, capturing increasing responses up to a plateau
Data requirements	Robust even with small or sparse datasets
Key outputs	Nutrient–response curves, derivative-based marginal efficiency, key nutritional metrics, Enhanced boxplots with post-hoc statistical comparisons for group differences
Statistical robustness	Bootstrap-derived confidence intervals; enhanced reliability of estimates
Implementation and availability	User-friendly, no-code application NutriCurvist; freely available for users

### Future directions

While NutriCurvist represents advancement, it is not without limitations. First, it assumes a monotonic, sigmoidal response—a reasonable assumption for essential nutrients^1,2^ but potentially inadequate for nutrients with toxicity thresholds or non-monotonic responses^29^. Future versions could extend the framework to handle non-monotonic or toxicological responses by incorporating modular architectures^9^ or hybrid interpretable components. The *tanh* function is highly flexible; however, it is not the only possible activation specifically when the response is not S-shaped. Future work could explore other interpretable activations or allow users to select from a small library of biologically plausible functions. Second, while data augmentation simulates within-group variability, it does not address between-study heterogeneity in meta-analyses. For such applications, hierarchical Bayesian extensions of NutriCurvist could be developed. Third, the current framework models one nutrient at a time. Real-world nutrition is multidimensional. A natural extension would be a “NutriCurvist-2D” for N–R interactions, using a two-neuron network with interpretable interaction terms.

## Conclusion

A single artificial neuron with a *tanh* activation function provides a simple yet interpretable framework for modeling N–R relationships across diverse datasets. This minimalist approach, comparable in complexity to a 4-parameter logistic model, successfully balances flexibility and nutritional interpretability. It produces robust estimates of key nutritional metrics, including nutrient requirements, even from small datasets. The approach is implemented in the user-friendly, no-code application NutriCurvist, facilitating adoption in applied nutrition research.

### Software availability

NutriCurvist is provided as a freely available, no-code graphical application. The software is openly hosted at Zenodo (DOI: 10.5281/zenodo.17184212) for permanent citation^30^. The latest version and ongoing development are also accessible via GitHub: https://github.com/hahmadima/NutriCurvist_ver_01. A detailed guide for installation and use of the application is provided in Supplementary Information.

## Supplementary Information

Below is the link to the electronic supplementary material.

Supplementary Material 1

## Data Availability

Software availabilityNutriCurvist is provided as a freely available, no-code graphical application. The software is openly hosted at Zenodo (DOI: 10.5281/zenodo.17184212) for permanent citation^30^. The latest version and ongoing development are also accessible via GitHub: https://github.com/hahmadima/NutriCurvist_ver_01. A detailed guide for installation and use of the application is provided in Supplementary Information.

## Declarations

### Further information

Presented in part: Ahmadi H, and Rodehutscord M. 2025. Machine learning-driven modeling of nutrient-response curves applied to a broiler chicken protein accretion dataset (abstract). 24th European Symposium on Poultry Nutrition. Maastricht, the Netherlands.
