# Supplementary material for: Nutrient–response modeling with a single and interpretable artificial neuron
Source: Sci Rep. 2025 Nov 24;15:41836. doi: 10.1038/s41598-025-29267-w (PMC12647789; doi:10.1038/s41598-025-29267-w)
Supplement: Supplementary file 1 — Supplementary Material 1 [file 41598_2025_29267_MOESM1_ESM.docx]

**📘 How to Install and Run NutriCurvist 1.0 App**

(No Coding Required!)

**1. Download the App:**

NutriCurvist 1.0 is freely available from Zenodo ([DOI: 10.5281/zenodo.17184212](https://doi.org/10.5281/zenodo.17184212)) or GitHub (<https://github.com/hahmadima/NutriCurvist_ver_01>).

**2. Extract the Files:**

After downloading, **unzip or extract** the file

**3. Install the MATLAB Runtime (One-Time Setup):**

Open the folder: NutriCurvist_ver_01 > MATLAB_Runtime and locate the installer file: **MyAppInstaller_web.exe**

Double-click it to begin installation.

🔄 This will:

- Automatically download and install MATLAB Runtime
- Require a **stable internet connection**
- Use around **2 GB of free disk space**
- Take a few minutes depending on your internet speed

💡 *If the installer doesn’t start, try* **Right-click → Run as Administrator**

Follow the on-screen instructions to complete the setup. This is a **one-time installation**. You won’t need to install the Runtime again for future versions of NutriCurvist.

**4. Launch the App:**

After installing the Runtime,
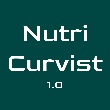
go back to the main folder **Double-click NutriCurvist.exe** to start the app. You're ready to use NutriCurvist!

**Compatibility Note:** NutriCurvist 1.0 has been successfully tested with MATLAB versions 2023a to 2025a and using the standalone Runtime (MCR). If your installed version is within this range or you use our provided Runtime, the app should work without any problems. It has also been tested on several regular desktop and laptop computers, and it runs smoothly even with standard processing power and memory.

## Getting Started with NutriCurvist 1.0

## 📄 Data

NutriCurvist 1.0 uses a **built-in data entry table**

Use the **"Data Entry"** panel on the right side of the main window.
• Manually type or **paste** your data into the table.
• Each row represents one observation (replicate)

You can manage the table by right-clicking to paste data, add or remove rows, or use **Ctrl+V** to paste directly.

**💡 Tips:**

For the analysis to run correctly, **every cell must have a valid entry**:
• No empty cells
• No missing or invalid numbers
• No text in numeric columns (X and Y)
• The first column (Sample_name) must contain at least one character, such as A1, A2, Sample1, or Sample2

If cells are empty or invalid, the app will either:
▪ Show an error message, or
▪ Return inaccurate or failed calculations.

**✅ Best Practices**

• Use **at least 5 levels of nutrient and 4 replicates per group** for better modeling accuracy
• Use the **"Clear Data"** button to reset the table
• Always check your table for complete, valid entries before clicking **"Run Analysis"**

**User guide for NutriCurvist**

When starting **NutriCurvist.exe**, a window (Figure 1) will appear where the user is required to enter several values before running the model. These values are necessary for customizing the neural network and data processing pipeline for your specific dataset.


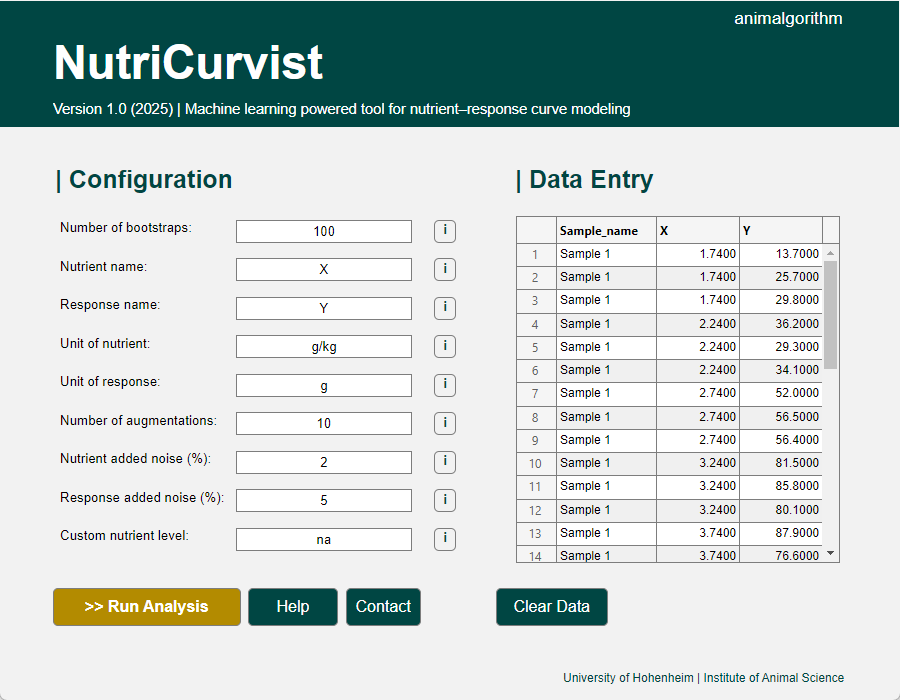


**Figure 1.** User interface of the NutriCurvist

Below is a description of each input field, what it means, what to enter, and how it is validated.

1. Number of Bootstraps
   • **What to enter**: A positive integer ≥ 10 (e.g., 100, 200)
   • **Meaning**: Determines how many random resampled datasets are created to ensure robustness and uncertainty estimation.
   • **Validation**: Must be a whole number (integer); 10 or higher
2. Nutrient name
   • **What to enter**: A text string (e.g., "Lysine", "Crude Protein")
   • **Meaning**: The name of the nutrient input variable; used as the X-axis label in plots.
   • **Validation**: Cannot be left empty
3. Response name
   • **What to enter**: A text string (e.g., "Weight Gain", "Egg Output")
   • **Meaning**: Name of the response variable; used as the Y-axis label in plots.
   • **Validation**: Cannot be left empty
4. Unit of nutrient
   • **What to enter**: A text string (e.g., "%", "g/kg")
   • **Meaning**: The unit of measurement for the nutrient; shown in axis labels.
   • **Validation**: Cannot be left empty
5. Unit of response
   • **What to enter**: A text string (e.g., "g/day", "mg/egg")
   • **Meaning**: Unit for the response variable.
   • **Validation**: Cannot be left empty
6. Number of augmentations
   • **What to enter**: A non-negative integer (e.g., 0, 10, 50)
   • **Meaning**: Number of extra synthetic data points to generate using noise-added copies of your original data. Useful when working with limited datasets to improve model robustness. Set to 0 if you have sufficient data.
   • **Validation**: Must be 0 or higher
7. Nutrient added noise (%)
   • **What to enter**: A non-negative number (e.g., 0, 2, 5)
   • **Meaning**: Amount of random noise (as a % of each original nutrient value) to be added to the nutrient-values for augmentation. Only applies if augmentation > 0.
   • **Validation**: Must be a non-negative number. If Number of augmentations is 0, then this must also be 0
8. Response added noise (%)
   • **What to enter**: A non-negative number (e.g., 0, 5, 10)
   • **Meaning**: Amount of random noise (as a % of each original response value) to be added to the response-values for augmentation. Only applies if augmentation > 0.
   • **Validation**: Must be a non-negative number. If Number of augmentations is 0, then this must also be 0
9. Custom nutrient level
   • **What to enter**: A non-negative number (e.g., 12.5) or the text 'na' to skip
   • **Meaning**: Allows scenario testing at a specific nutrient value. When set, the model evaluates the response at this nutrient level.
   • **Validation**: Must be a non-negative number or the text 'na'. If 'na' is entered, the analysis at a custom nutrient level is skipped

## ****Results****

Once NutriCurvist finishes analyzing your data and modeling is complete, the software automatically generates a results window with the following items inside:

This window contains **4 sheets**:

1. **Metrics**
   - Includes metrics (e.g., R², RMSE, nutrient required to reach desirable response, etc.) from the curve fitting process for each sample including their 95% confidence intervals.
2. **Equations**
   - Contains the final symbolic formulas (ANN-based) fitted for each dataset. These are useful for interpretation or further use.
3. **Coefficients**
   - Includes the estimated neural network parameters (weights and biases) for each sample:
   - Each parameter also includes its **95% confidence intervals**, based on bootstrapping.
4. **Data & predictions**

### Figures: Plots and metrics comparison

### A save option is included in all sheets and Figures.

**Insight from Results**

The basic model used in **NutriCurvist** is a simple artificial neural network with **one artificial neuron** and a **tanh activation function**, designed to fit smooth and meaningful in the context of nutrient–response analysis (Figure 1 and Table 1).

**Model Equation:** The fitted model takes the following mathematical form:

$Response=\mathbf{A}\tanh\left( \mathbf{c} Nutrient+\mathbf{b} \right)+\mathbf{B}$ Equation 1





**Figure 2:** Schematic nutrient–response curve generated by the Equation 1, with parameters A = 50, c = 0.05, b = -1.1, and B = 30. For definitions see Table 1.

**Table 1.** Definition of NutriCurvist model, parameters, and derived nutritional metrics.

|  | **Explanation** | **Equation** |
| --- | --- | --- |
| **Model** | Nutrient–response curve describing how response changes with nutrient | $Response = A\tanh\left( c Nutrient + b \right)+ B$ |
| **First derivative** | Rate of change of the response with respect to nutrient | $\frac{\mathrm{dResponse}}{\mathrm{dNutrient}}=A c \left( 1-{tanh}^{2} \left( c Nutrient+b \right) \right)$ |
| **Parameters** |  |  |
| $A$ | Amplitude of the curve determines how much the response increases beyond the inflection point |  |
| $c$ | Steepness parameter: controls how quickly the response changes as nutrient level increases |  |
| $b$ | Horizontal translation: shifts the curve left or right along the nutrient axis |  |
| $B$ | Response at the inflection point; with B ≈ 0, the curve rises symmetrically from the origin toward the plateau |  |
| **Nutritional metrics** |  |  |
| $r_{\max}$ | Maximum slope of the curve; the steepest rate of increase in response per unit nutrient at the inflection point | $A c$ |
| $\mathrm{Nutrient}^{*}$ | Nutrient level at the inflection point, where response changes most rapidly | $-\frac{b}{c}$ |
| $\mathrm{Response}^{*}$ | Response value at the inflection point (i.e. $\mathrm{Nutrient}^{*}$) | $B$ |
| $\mathrm{Nutrient}_{\mathrm{Lag}}$ | Lowest nutrient value where a meaningful response begins ($\mathrm{Response}_{\min}=$ model-predicted response at lowest nutrient) | $\mathrm{Nutrient}^{*}-\frac{\mathrm{Response}^{*}-\mathrm{Response}_{\min}}{r_{m}}$ |
| $Half-life$ | Nutrient level at which 50% of the asymptotic response is reached | $\frac{arctanh \left( 0.5-0.5\frac{B}{A} \right)- b}{c}$ |
| $\mathrm{Response}_{\infty}$ | Asymptotic response as nutrient increases indefinitely | $A + B$ |
| $\mathrm{Req}_{95\%}$ | Nutrient needed to achieve 95% of the $\mathrm{Response}_{\infty}$ | $\frac{arctanh \left( 0.95-0.05\frac{B}{A} \right)- b}{c}$ |
| $\mathrm{Req}_{99\%}$ | Nutrient needed to achieve 99% of the $\mathrm{Response}_{\infty}$ | $\frac{arctanh \left( 0.99-0.01\frac{B}{A} \right)- b}{c}$ |
